# Genomic and Proteomic Analysis of *Schizaphis graminum* Reveals Cyclophilin Proteins Are Involved in the Transmission of *Cereal Yellow Dwarf Virus*

**DOI:** 10.1371/journal.pone.0071620

**Published:** 2013-08-09

**Authors:** Cecilia Tamborindeguy, Michael S. Bereman, Stacy DeBlasio, David Igwe, Dawn M. Smith, Frank White, Michael J. MacCoss, Stewart M. Gray, Michelle Cilia

**Affiliations:** 1 USDA-ARS, Robert W. Holley Center for Agriculture and Health, Department of Plant Pathology and Plant-Microbe Biology, Cornell University, Ithaca, New York, United States of America; 2 Department of Genome Sciences, University of Washington, Seattle, Washington, United States of America; 3 Virology and Molecular Diagnostics Unit, International Institute of Tropical Agriculture, Ibadan, Nigeria; 4 Department of Plant Pathology, Kansas State University, Manhattan, Kansas, United States of America; Volcani Center, Israel

## Abstract

Yellow dwarf viruses cause the most economically important virus diseases of cereal crops worldwide and are transmitted by aphid vectors. The identification of aphid genes and proteins mediating virus transmission is critical to develop agriculturally sustainable virus management practices and to understand viral strategies for circulative movement in all insect vectors. Two cyclophilin B proteins, S28 and S29, were identified previously in populations of *Schizaphisgraminum* that differed in their ability to transmit the RPV strain of *Cereal yellow dwarf virus* (CYDV-RPV). The presence of S29 was correlated with F2 genotypes that were efficient virus transmitters. The present study revealed the two proteins were isoforms, and a single amino acid change distinguished S28 and S29. The distribution of the two alleles was determined in 12 F2 genotypes segregating for CYDV-RPV transmission capacity and in 11 genetically independent, field-collected *S* *. graminum* biotypes. Transmission efficiency for CYDV-RPV was determined in all genotypes and biotypes. The S29 isoform was present in all genotypes or biotypes that efficiently transmit CYDV-RPV and more specifically in genotypes that efficiently transport virus across the hindgut. We confirmed a direct interaction between CYDV-RPV and both S28 and S29 using purified virus and bacterially expressed, his-tagged S28 and S29 proteins. Importantly, S29 failed to interact with a closely related virus that is transported across the aphid midgut. We tested for *in vivo* interactions using an aphid-virus co-immunoprecipitation strategy coupled with a bottom-up LC-MS/MS analysis using a Q Exactive mass spectrometer. This analysis enabled us to identify a third cyclophilin protein, cyclophilin A, interacting directly or in complex with purified CYDV-RPV. Taken together, these data provide evidence that both cyclophilin A and B interact with CYDV-RPV, and these interactions may be important but not sufficient to mediate virus transport from the hindgut lumen into the hemocoel.

## Introduction

Barley and cereal yellow dwarf viruses (B/CYDV) in the genera *Luteovirus* and *Polerovirus* that cause yellow dwarf disease of monocots are phloem restricted, single-stranded positive-sense RNA plant viruses [1]. They are strictly dependent on aphid vectors for host-to-host transmission, and they are transmitted in a circulative, non-propagative manner [2]. The model for circulative transmission involves aphids ingesting virions while feeding on the phloem sap of infected plants. Virions are acquired into the vector by moving through the hindgut cells and then are released into the hemocoel. Virions circulate in the hemolymph and concentrate at the basal lamina of the accessory salivary gland. Virions are then actively transported across these cells and released into the salivary duct where they can be injected, along with salivary secretions, as the aphid feeds on a plant host. Luteo and poleroviruses causing yellow dwarf disease are believed to follow the circulative pathway through aphid vectors [2]; however, virus transmission is aphid-species specific. All aphids can ingest the various viruses during phloem feeding, but only some of the viruses are transmitted by any single aphid species [3]. Transmission will not occur if the virus fails to cross one of two potential transmission barriers; the hindgut or the accessory salivary gland. Virus particles are transported across both tissue types by a mechanism that resembles receptor-mediated endocytosis [4], with different ligands and receptors involved at each step. Viruses in the *Luteoviridae* do not replicate in their vector and are transmitted only as virus particles [5]. The virus capsid contains two viral proteins: a 22 kDa major coat protein (CP) and a minor 72 kDa read-through protein (RTP) [6]. These are the only viral proteins required for transmission, and both are required to interact with aphid components to facilitate virus transport through the aphid [7–11]. Chemical cross-linking coupled to mass spectrometry revealed a distinct topological feature in the a polerovirus RTP that is required for virus-aphid interactions [11].

Little is known about the aphid components responsible for virus transmission. Several candidate proteins have been identified using virus overlay assays [12–14] and more recently proteomic approaches [15–17]. Two proteins (SaM35 and SaM50), able to bind *in vitro* the MAV strain of Barley yellow dwarf virus (BYDV), were isolated from the vector *Sitobion* *avenae* , but they were not detected in the nonvector aphid *Rhopalosiphummaidis* [12]. Another 50 kDa protein, able to bind to the GAV strain of BYDV, was detected in two vector species *Schizaphisgraminum* and *S* *. avenae* , but not in the nonvector species, *Rhopalosiphumpadi* [14]. None of these proteins were identified using mass spectrometry. Three *Myzus* *persicae* proteins that bound the related *Beet western yellows virus* were identified by mass spectrometry as actin, a receptor for activated C kinase 1 (Rack-1), and Glyceraldehyde-3-phosphate dehydrogenase 2 (GAPDH) [13]. Rack 1 has been shown to be involved in the regulation cell surface receptors [18] and GAPDH is an enzyme of the glycolysis pathway that also regulates endocytosis when phosphorylated [19]. Actin is involved in intracellular trafficking, it interacts with endocytic components [20] and is involved in virus transport [21]. These proteins may play a role in virus transmission, but no direct evidence was provided and the protein interaction experiments were performed under denaturing conditions.

Validating the involvement of proteins in the circulative transmission process has posed a significant challenge to the entire vector biology field. Aphids are not amenable to transgenesis. Functional analyses are possible but difficult to achieve. RNA silencing pathways are conserved and even expanded in aphids [22]. RNA interference (RNAi) has been successful [23,24] although silencing is incomplete in these insects. Dissected gut and salivary gland tissues are tiny, making biochemistry studies difficult. No suitable aphid cell culture models are available to study protein function. However, aphids are an ideal vector species to couple genetics and proteomics to probe protein function [15–17]. Aphids are cyclic parthenogens; they alternate sexual reproduction with parthenogenetic reproduction. Aphid hybrid lineages generated by sexual reproduction can be maintained parthenogenetically and allow the investigator to phenotype each hybrid genotype for different traits, for instance transmission of different virus strains or virus species. Parthenogenetic reproduction makes aphids highly amenable to proteomics studies because massive quantities of protein can be generated from genetically identical aphids as easily as growing bacterial cultures. Furthermore, the genome sequence of the pea aphid *Acyrthosiphon pisum* has been published [25] information on key pathways regulating the genetic basis of phenotypic plasticity [26,27] and the aphid EST collection is also expanding.

To help dissect the mechanism of transmission of luteo and poleroviruses, an *S* *. graminum* population was developed by crossing two genotypes that differed for their ability to vector the RPV strain of *Cereal yellow dwarf virus* (CYDV-RPV) and the SGV strain of *Barley yellow dwarf virus* (BYDV-SGV) [28]. These genotypes do not harbor known secondary endosymbionts, only distinct genotypes of the primary endosymbiont *Buchnera* spp [16]. The F2 aphid genotypes were characterized for their ability to transmit both viruses. It was found that the ability to transmit each virus segregated independently in the population [29]. Moreover, it was found that the barrier (i.e. hindgut or accessory salivary gland) responsible for preventing virus movement in the nonvector genotypes also segregated; some of the nonvector F2 genotypes had a strong hindgut barrier, others had a strong accessory salivary gland barrier and others had both barriers.

Proteomic studies of this aphid population identified several proteins that were differentially expressed between vectors and nonvectors [16,17]. Among these proteins were two cyclophilins (proteins S28 and S29) identified by 2-D fluorescence difference gel electrophoresis (DIGE) coupled to mass spectrometry to have similarity to the protein encoded by the *Acyrthosiphon pisum* EST gi 82571971 [17]. The S28 protein was present in all eight genotypes analyzed whereas the S29 protein was only found in protein extracts from the four vector genotypes analyzed. Based on 2-D DIGE, the two proteins had similar molecular weights, but slightly different pIs. A protein of similar molecular weight and pI to S29 was detected following incubation of a total aphid protein extract with purified CYDV-RPV and a virus-specific antibody [17] using a co-immunoprecipitation (co-IP)-DIGE approach; however, this protein was not identified using mass spectrometry.

Cyclophilins are peptidyl-prolyl isomerases proteins (PPIases) [30]. They catalyze the isomerization of peptide bonds from trans form to cis form at proline residues and they facilitate protein folding. The cyclophilins identified by Yang et al. (2008) were related to the *Drosophila melanogaster* CG2852 protein (NP_611695) and to the human Cyclophilin B protein (NP_000933). Cyclophilin B is localized to the endoplasmic reticulum and extracellular space [31] as well as on the cell surface of mammalian cells [32]. This protein functions in the secretory pathway, possibly by chaperoning membrane proteins or having a role in receptor signaling pathways [33]. A direct role of cyclophilin in B/CYDV movement through aphids may involve chaperoning the virus to various membrane bound vesicles, e.g. endosomes, in either gut or salivary tissues. In this study, we used a combination of genomic, proteomic, and biological approaches to probe the function of cyclophilin in virus transmission.

## Materials and Methods

### Aphid Genotypes and Virus Strains

Virus-free genotypes of *S* *. graminum* [28] were maintained parthenogenetically as described previously [34]. Additional biotypes of *S* *. graminum* were obtained from Dr. John Burd, USDA, ARS, Still water, OK. Aphid biotypes were determined based on their ability to infest different host plants [35]. CYDV-RPV and the related potato leafroll virus (PLRV) were maintained and purified as described previously [17,36].

### Virus Transmission Assays

Aphids were allowed a 48 h acquisition access period (AAP) on leaves detached from BYDV-RPV-infected plants inoculated 4 to 5 weeks previously. Viruliferous aphids were transferred from the virus source leaves to 12 recipient noninfected ‘Coast Black’ oat plants and allowed a 5 day inoculation access period (IAP). Plants were observed for symptom development for 3 to 5 weeks. Virus transmission efficiency was calculated as the percentage of the total number of plants infested with viruliferous aphids that become infected.

### Cyclophilin Sequencing

RNA from each aphid genotype and biotype was extracted using the RNeasy Plant Mini Kit (QIAGEN). The RNA was reverse transcribed using the SuperScript® First-Strand Synthesis System for RT-PCR (Invitrogen). Cyclophilin primers amplifying the complete coding sequence of the *A* *. pisum* gi 82571971 EST were designed (F5’ATGATATCTACTTATAAAATCATGACG3’ and R5’TTATTCGGTAGCATCAGTTTTG3’). The PCR conditions consisted of a denaturation step at 95^°^ C for 2 min, 30 cycles at 95^°^ C for 15 sec, 55^°^ C for 30 sec and 72^°^ C for 1 min, and a final extension step at 72^°^ C for 5 min. The PCR products were purified using the QIAquick PCR Purification Kit (QIAGEN) and sequenced using each one of the cyclophilin primers.

### In Silico Analysis

*In silico* analyses were performed using the ExPASy World Wide Web server (http://ca.expasy.org/tools/pi_tool.html) [37].

### Statistical Analyses

The Wilcoxon rank-sum test using R software was applied to test differences in CYDV-RPV transmission efficiency between genotypes encoding or not the cyclophilin vector allele. P values less than 0.05 were regarded as statistically significant.

### Cyclophilin *In vitro* Binding Assay

Both forms of *S* *. graminum* cyclophilin were cloned in the expression vector pET101/D-TOPO and expressed in *Escherichia coli* using the Champion™ pET Directional TOPO® Expression Kit (Invitrogen). The expressed proteins were purified under native conditions using the Ni-NTA Fast Start kit (Qiagen). Co-immunoprecipitation (co-IP) between the expressed cyclophilin proteins and purified CYDV-RPV or PLRV was performed as described in Yang et al. (2008), with the exception that following the washes the co-immunoprecipitated proteins were kept in 0.025 M phosphate-buffered saline containing 0.15 M NaCl (pH 7), boiled in Laemmli buffer and separated on a 12% SDS-PAGE gel. The proteins were transferred onto Immobilon-P membranes (Millipore) using a semi-dry transfer apparatus (Thermo Scientific) for 95 min at room temperature according to the manufacturer’s instructions. Filters were incubated 1 h at room temperature with gentle shaking in TTBS (100 mM Tris, 0.9% NaCl, 0.1% Tween) with a 1/1000 dilution of Penta·His Antibody (QIAGEN). After brief washes in TTBS, filters were incubated for 1 h at room temperature with gentle shaking in TTBS with a 1/5000 dilution of goat anti-mouse IgG alkaline phosphatase (AP) conjugated (Sigma). Filters were subsequently washed in TTBS and proteins were detected by the addition of 1-Step NBT/BCIP (Pierce) as per the manufacturer’s instructions.

### Virus and Aphid Protein Co-immunoprecipitation

To quantify interactions between cyclophilin and CYDV-RPV, we performed a co-immunoprecipitation (co-IP) experiment coupled to a bottom-up LC-MS/MS analysis. Aphid tissue (2 g of each aphid genotype, two vectors, A3 and WY10-A, and one nonvector C2) were placed into a pre-chilled mortar and covered with liquid nitrogen. Aphid proteins were extracted into 2 mL of 0.1 M phosphate buffer pH 6.7 containing 1% EDTA-free HALT protease inhibitors (Pierce, Rockford, IL) and clarified as described [17]. Partially purified CYDV-RPV was prepared as described [17] with the following modifications. Virus pellets were recovered following a 2 h centrifugation at 40,000 x G in a 30% sucrose cushion and resuspended in 0.1 M phosphate buffer, pH 6.7. Virus was stored at -80° for 24 hr, thawed on ice, and quantified using a Nanovue spectrophotometer (GE Healthcare, Piscataway, NJ). 200 µg of partially purified virus solution was added to each protein extract and rotated at 4° C for 6 hr. An additional reaction containing proteins extracted from biotype WY-10A was used as a negative control with no virus added to enable us to pinpoint the highly abundant aphid proteins that interact with antibodies or beads from further consideration.

Dynal m270 epoxy beads (Life Technologies, Carlsbad, CA) were conjugated overnight at 37° to anti-CYDV-RPV antibodies at an antibody:bead ratio of 10 µg antbody:10 μg beads according to the Cristea and Chait protocol [38]. Aphid-virus protein complexes were added to the conjugated beads for a total volume of 2.2 mL per reaction. Protein complexes were co-immunoprecipitated for 12 hr at 4° C. Magnetic beads were washed six times in a 0.025 M phosphate buffer containing 0.15 M NaCl to remove loosely and unbound proteins. The tubes were changed following each wash to eliminate proteins that nonspecifically bound to the plastic from the elution. Protein complexes were eluted in 0.5 N NH_4_OH and 0.5 mM EDTA, flash frozen in liquid nitrogen and dried using a vacuum centrifugal concentrator. Protein complexes were resuspended in 8 M urea in 100 mM NH_4_HCO_3_. Proteins were reduced using 10 mM DTT, and thiols were sulfenylated using 30 mM methyl methanethiosulfonate. Proteins were hydrolyzed into peptides using trypsin (Promega, Agora, WI) for 12 h. Salts and impurities were removed using mixed mode strong cation exchange reversed phase cartridges (Waters Oasis 1cc MCX cartridge).

### LC-MS/MS

Each aphid genotype co-IP and control co-IP was analyzed in triplicate. The control co-IP was subjected to the same LC-MS/MS methods as the experiment coIP with virus. Co-IP and control runs were randomized to eliminate any potential artifacts introduced due to run order. Split-less nanoflow chromatography was performed in the vented column configuration using a Waters NanoAcquity LC system (Waters Corporation, Milford, MA). Peptides were reconstituted in 30 µl solvent A. Solvents A and B were 99.9/0.1 water/formic acid and 99.9/0.1 acetonitrile/formic acid, respectively. A flow rate of 2 µL/min (98% A/2% B) flushed sample out of a 5 µL loop and onto a self-packed capillary trap column (100 µm ID × 4 cm). After 10 µL of wash, the six-port valve switched and closed the vent, which initiated the gradient flow (250 nL/min) and data acquisition. A 70 min analysis was used in which solvent B ramped from 2% to 32% over 43 min (2-45 min); from 32% to 80% over 1 min (45-46 min); held constant for 5 min (46-51 min); and then initial conditions were restored (51-52 min) and held constant for the final 18 min (52-70 min)

A Q-Exactive (Thermo, Fisher, Bremen, Germany) was operated in data dependent mode for mass spectrometric analysis. For each precursor scan, the top 12 most abundant ions were selected for tandem MS. For MS1 scans, a resolving power of 35000 at *m/z* 200 was used with an automatic gain control (AGC) of 1,000,000 charges and a max ion injection time (IT) of 10 ms. A resolving power of 17,500 at *m/z* 200 was set with an AGC of 200,000 charges and a max IT time of 55 ms for MS2 analysis. A 90 s exclusion window was used to avoid repeated selection of abundant ions. For selection of ions, peptide-like isotope distributions were preferred with the exclusion of unassigned and 1^+^ charge states.

### Database Searching

Tandem mass spectra were converted into mascot generic format (MGF) peak list files using msconvertGUI available from Proteowizard (http://proteowizard.sourceforge.net/tools.shtml) [39]. An initial database search of all insect, plant, and bacterial proteins in NCBI revealed the presence of only one plant protein in co-IP datasets. Thus, to increase the coverage of *S* *. graminum* proteins, we researched the data using an in-house database created from a collection of 454 sequencing products generated from *S* *. graminum* biotype H pooled head and gut mRNA libraries. The genome sequence of CYDV-RPV and cyclophilin B sequences were added to the database. In total, the database had 297,312 sequences. The cyclophilin mRNA sequence is provided here (Figure S1), and all sequences are available in the NCBI Short Read Archive: http://www.ncbi.nlm.nih.gov/biosample/2265610. All data were searched using Mascot v 2.3.02 (Matrix Science, Boston, MA) as follows. Fixed methylthio on cysteine residues and variable methionine oxidation and deamidation of asparagine and glutamine were used as modifications. The precursor mass measurement accuracy tolerance was set to 30 ppm, and fragment ion tolerance was 0.2 Dalton (Da). Instrument type was not specified. A single missed tryptic cleavage was permitted.

### Label-free Quantification

Two methods of label-free quantification were used, spectral counting and MS1 peak area comparisons, to investigate whether cyclophilin was enriched in the co-IP as compared to the control co-IP with no virus. For spectral counting, Mascot *. dat files were created in Mascot and loaded into Scaffold (version 3_00_05). Peptide and protein probabilities were calculated using PeptideProphet and ProteinProphet algorithms [40]. Protein and peptide FDR was 0.0%. Spectral counts were normalized to the total and compared between co-IP with virus and no virus control. A Fisher’s Exact Test was performed to test for spectral count differences between groups. We used Skyline [41] to perform label-free quantification of MS1 ion signals derived from cyclophilin peptides in the co-IP [42]. Skyline is an open source software program that can be downloaded from http://proteome.gs.washington.edu/software/skyline. We created a comprehensive spectral library including sampling across multiple acquisitions in Skyline that contains all the MS/MS spectra from the co-IP experiments. Search engine parameters were adjusted in Skyline as described above for Mascot searches. The precursor isotopic import filter was set to a count of three (M, M+1, M+2) at a resolving power of 35,000 at 200 m*/z*. Raw files were then imported directly into Skyline. Extracted ion chromatograms were manually inspected for each peptide. A normalization factor was calculated to account for run-to-run variation in ion abundances. Peak areas from three peptides derived from a protein that showed no enrichment in the co-IP as compared to the healthy control were normalized to the total. A normalization factor was calculated for each peptide. These normalization factors were averaged for each individual LC-MS/MS run. Total peak areas for the cyclophilin peptides of interest from every MS run were normalized by the average normalization factor. Raw and normalized peak areas for the cyclophilin peptides can be found in Dataset S1. A Kruskal-Wallis test was performed to investigate differences between the normalized peak areas for each peptide.

## Results

### Identification of the Transcript Sequences for Both Cyclophilin Proteins

Cyclophilin cDNA was initially amplified from both parent genotypes of the *S* *. graminum* population [28] used to first identify the two cyclophilin proteins. A single 660 bp product was amplified from each genotype and subsequently sequenced. Two transcript forms were identified, one from each parent that differed by two nucleotides and that were 90% similar to the *A* *. pisum* EST gi 82571971. The first difference between the parent transcripts involved a single nucleotide polymorphism (C to G substitution) at position 95 that results in an amino acid change. The vector parent encodes a glutamine residue (Q) whereas the nonvector parent encodes glutamic acid (E). The second difference involved an A to T substitution at nucleotide 406 that does not result in an amino acid change.

*In silico* analyses of the proteins encoded by each parent determined that both protein isoforms were similar in molecular weight, but slightly different pIs; the predicted pI for the isoform encoded by the vector parent was more basic than the predicted pI from the nonvector isoform (Table 1). A signal peptide predicted to be cleaved is present in both proteins and the differing amino acid is located in position 2 of the mature forms.

**Table 1:** Comparison of the observed and the predicted pI and molecular mass for both cyclophilin isoforms.

	Observed pI 2D DiGE	Predicted pI from sequenced cDNA	Observed mass 2D DiGE	Predicted mass from sequenced cDNA
S29/ CV	9	9.2	26.6	24.2
S28/ CNV	8.6	9.06	26.6	24.2

The observed data is based on the 2D-DiGE analysis published previously [17]. The predicted data was obtained by *in silico* analysis of the sequenced cDNAs using the Expasy Compute pI/Mw tool (http://ca.expasy.org/tools/pi_tool.html). The difference in mass could be a result of a posttranslational or co-analytical modification or simply because SDS-PAGE is not an accurate way to measure molecular mass.

### Cyclophilin cDNA Sequencing from F2 Genotypes Differing in Their Transmission Efficiency

The cyclophilin cDNA was sequenced from 12 F2 genotypes of the *S* *. graminum* population that differ in their CYDV-RPV transmission efficiency (Table 2). Three genotypes are efficient vectors and nine genotypes are inefficient vectors. The presence of each allele was assessed using the two differing nucleotides in position 95 and 406: the vector allele was characterized by a C in position 95 and an A in position 406.

**Table 2:** Transmission efficiency of CYDV-RPV by analyzed F2 genotypes of *S* *. graminum* aphids and the encoded Cyclophilin alleles.

Genotype	Transmission efficiency	Barrier	Cyclophilin alleles encoded
Vector parent	92%	None	Vector allele
Nonvector parent	0%	Salivary gland and gut	Nonvector allele
A3	100%	None	Vector allele
CC6	75%	None	Vector allele
G11	83%	None	Vector allele
BB1	0%	Salivary gland	Vector and Nonvector allele
C2	0%	Gut	Nonvector allele
CC1	0%	Gut	Nonvector allele
CC2	8%	Gut	Nonvector allele
CC5	0%	Gut	Nonvector allele
K2	0%	Salivary gland and gut	Nonvector allele
K3	0%	Salivary gland	Vector and Nonvector allele
LL3	0%	Gut	Nonvector allele
MM1	0%	Gut	Nonvector allele

Transmission efficiency is calculated as the number of plants infected with virus out of the number of plants infested with viruliferous aphids (5 aphids per plant, 12 plants used). Determination of transmission barriers in genotypes with low transmission efficiency is described in [28].

The analysis of the cyclophilin cDNA sequence of the three vector genotypes tested (A3, CCS 6 and G11) indicated that both alleles were present in these genotypes. Among the nine F2s that transmit CYDV-RPV less efficiently, seven (C2, CC1, CC2, CCS 5, K2, LL3 and MM1) were found to only possess the nonvector allele, whereas the other two genotypes (K3 and BB1) possessed both forms (Table 2). We tested the influence of the presence of the cyclophilin vector allele on the transmission efficiency of the *S* *. graminum* genotypes. The transmission efficiencies were significantly higher for the genotypes encoding the cyclophilin vector allele (Wilcoxon rank-sum test, p=0.04545 using only the F2 genotypes and p=0.01449 if the parental genotypes were included).

### Analysis of the Cyclophilin Gene in Different S *graminum* Biotypes

Eleven distinct *S* *. graminum* biotypes (NY, B, C, E, F, G, H, I, K, Ks, Flo) collected in the field and characterized by the Burd Lab [43] were analyzed to determine if there was a correlation between the presence of the vector allele of the cyclophilin gene and the ability to transmit CYDV-RPV among wild populations of the aphid.

**Figure 1:**
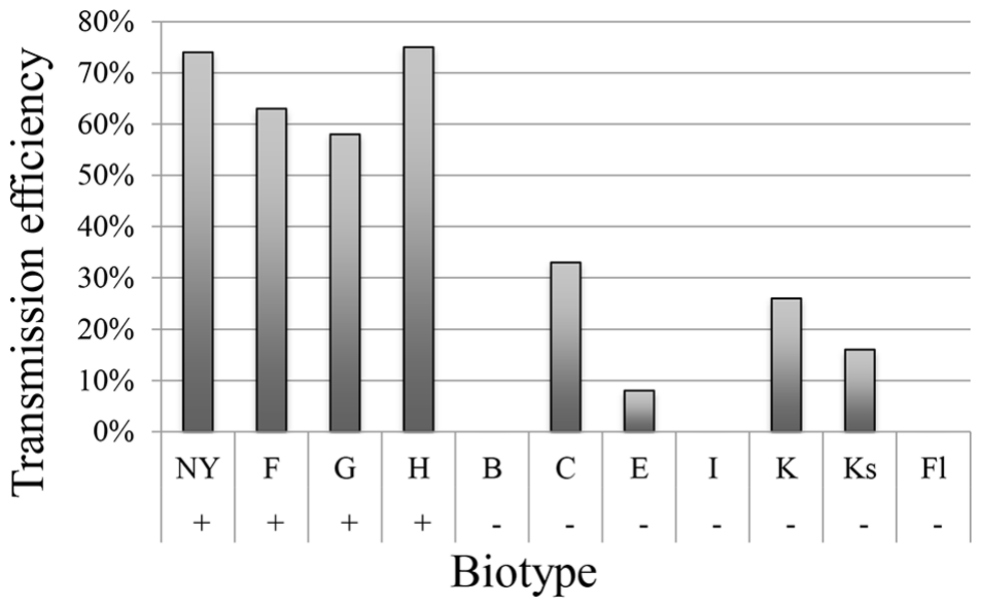
Transmission efficiency is correlated to presence of vectoring cyclophilin allele in field-collected aphid biotypes. Transmission efficiency is calculated as the number of plants infected with virus out of the number of plants infested with viruliferous aphids (five aphids per plant, 12 plants used). +/- indicates the detection of the vectoring allele. Biotypes NY and H were heterozygous. Biotypes NY, F, G, and H efficiently transmitted CYDV-RPV whereas Biotyoes B, I, and Fl did not transmit at all. Biotypes C, K and Ks transmitted with poor efficiencies.

The NY population has been maintained in the Ithaca laboratory since the late 1950’s and is an efficient vector of CYDV-RPV [44]. In addition to the NY biotype, three biotypes, F, G and H were efficient vectors of CYDV-RPV, whereas the remaining seven biotypes transmitted CYDV-RPV with lower efficiency (Figure 1). The analysis of the sequence of the cyclophilin cDNA identified 5 cyclophilin alleles from the various biotypes: 1 encoded the vector isoform and 4 encoded the nonvector isoform (Figure S2). The four efficient vectors, NY, F, G and H, encoded the vector isoform whereas the seven less efficient vectors were homozygous for the nonvector allele (Figure 1). Statistical analysis showed the biotypes encoding the vector allele of cyclophilin transmitted CYDV-RPV with significantly higher efficiency than the biotypes encoding only nonvector alleles (Wilcoxon rank-sum test, p=0.006061).

### Direct Interaction of *Schizaphis graminum* Cyclophilin with Poleroviruses

**Figure 2:**
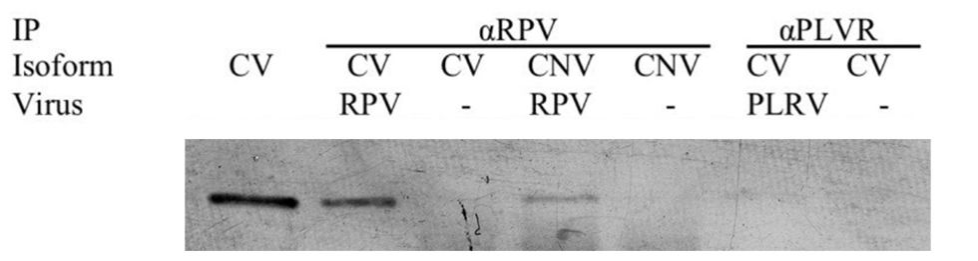
Interaction between the cyclophilin protein and CYDV-RPV and PLRV purified virus. His-tag cyclophilin vector (CV) and nonvector (CNV) isoforms were expressed *in vitro* in *E. coli* and co-immunoprecipitated with CYDV-RPV or PLRV. Co-immunoprecipitated proteins were detected with anti-his antibodies. First lane shows the synthesized cyclophilin protein. Interactions were notable between both isoforms and CYDV-RPV but not between PLRV and the vector isoform.

Next we tested ability the S28 and S29 cyclophilin isoforms to interact *directly* with purified CYDV-RPV virions. We evaluated this by *in vitro* co-immunoprecipitation between CYDV-RPV virions and cyclophilin proteins synthesized in *E. coli*. Both S28 and S29 were shown to interact with CYDV-RPV (Figure 2). The binding experiments are not quantitative and the amount of co-immunoprecipitated proteins is not easily compared between treatments. To determine if the *S* *. graminum* cyclophilin specifically binds to viruses transmitted by that aphid, the experiment was repeated using *Potato leafroll virus* (PLRV), a polerovirus related to CYDV-RPV, which is transmitted by *M* *. persicae* , but not by *S* *. graminum* . No definitive band was observed on the Western blot and a positive interaction between PLRV and *S* *. graminum* cyclophilin could not be validated.

**Figure 3:**
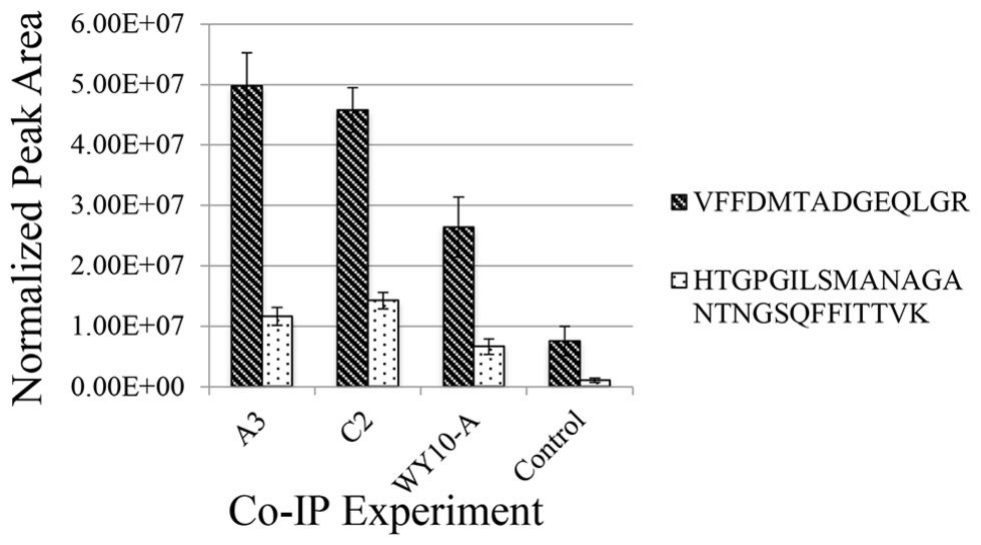
Normalized peak areas from two cyclophilin A peptides show enrichment in co-immunoprecipitation experiments using aphid proteins and purified CYDV-RPV. Whole insects of genotypes A3, C2 and biotype WY-10A (efficient vector biotype recently collected from a field in Wyoming) were subjected to cryogenic cell lysis and protein extraction. The extracted proteins were co-immonoprecipitated with purified CYDV-RPV using anti-RPV antibodies. Two peptides from cyclophilin were enriched in gentoypes A3, C2 and the field collected biotype WY-10A as compared to the control co-immunoprecipitation with no virus (aphid proteins incubated with beads and antibodies). Peptide FFDMTADGEQLR (2+ precursor *m/z* 793.369) was higher in intensity than the HTGPGILSMANAGANTNGSQFFTTVK peptide (3+ precursor *m/z* 912.871). This is not a good indicator of differences in relative abundance between the two peptides, which could result from different ionization efficiencies. However comparison of peak areas for each peptide across the various samples is an accurate way to measure relative abundance of the peptide in each sample. Both peptides showed similar trends in the experimental co-IPs compared to the control. Both peptides were more abundant in the co-IP reactions with virus. Although the peak areas showed an overall lower abundance in biotype WY10-A, which might reflect a lower overall expression of cyclophilin in this biotype as compared to the lab-reared F2 genotypes, this difference was not significant using a Kruskal Wallis test.

To perform a relative quantification of cyclophilin B to CYDV-RPV, we performed a bottom-up LC-MS-MS analysis of a co-IP between CYDV-RPV and a total aphid protein extract. Proteins were extracted from three genotypes of *S* *. graminum* proteins and the co-IP was performed as described in Yang et al. (2008). A full description of the aphid-RPV interactome will be described in a forthcoming manuscript. Surprisingly, cyclophilin B was not detected (data not shown). Instead, cyclophilin A was one of 30 proteins that were at least two-fold higher or unique in the co-IP reactions with virus compared to the negative control, p<0.05. Peptides specifically matching to cyclophilin A were detected in A3 and WY10A (vector genotypes) as well as C2, a nonvector genotype with only the nonvector allele (Dataset S2). Spectral counts were two-fold higher in the co-IP compared to the negative control indicating an enrichment of cyclophilin in the co-IP reactions with virus (Table 3). However, the spectral counts were not statistically different between vector (A3 and WY-10A) and the nonvector genotype (C2, Table 3). To further verify an enrichment of cyclophilin in the co-IP reactions, we compared the ion intensity chromatograms from the full scan MS data for two cyclophilin peptides that were selected for tandem MS and that had excellent retention time alignment across the replicates (Figure S3). The normalized total peak areas for these two peptides were significantly higher in the co-immunoprecipitation reactions as compared to the control reaction with no virus (Figure 3, P value = 0.022 and 0.027 using a Kruskal Wallis test, respectively), providing further support for an enrichment of cyclophilin A in the co-IP reactions. These data provide strong evidence that cyclophilin A also interacts either directly, or in complex, with CYDV-RPV.

**Table 3:** Cyclophilin spectral counts in the coIP with CYDV-RPV and *Schizaphisgraminum* proteins show more than 2-fold enrichment compared to negative control.

	Control			Vector WY10A			A3			Nonvector C2		
Replicate	1	2	3	1	2	3	1	2	3	1	2	3
Spectral Counts	0	2	4	6	5	6	1	7	5	3	7	5
Totals	6			17			13			15		

## Discussion

Proteomic and genomic analyses are not always in agreement due to biological, technical, or analytical factors. The proteomic and genomic analyses of the nonvector parent were in agreement; the S28 protein was the only protein observed [17] and the nonvector allele was the only transcript detected. The proteomic analysis [17] suggested the presence of two different cyclophilin B isoforms in the vector parent; however, the genomic results presented here indicate the vector parent was homozygous for the vector allele. A plausible explanation for the presence of two different protein isoforms in the proteomic analysis is the spontaneous deamination of glutamine that can occur in aqueous conditions [45]. The product of this deamination would be a protein of similar pI than the cyclophilin isoform present in the nonvector parent (S28). The spontaneous deamination of glutamine is a rate limiting reaction, and it could explain the difference in the amounts of both protein forms identified in the vector genotype where a much greater amount of the isoform S29 was detected when compared to the S28 [17]. A second explanation for this observation lies in the limitation of 2-D DIGE, the gel-based technology that was used to discover these isoforms. Using 2-D gels, a common problem is the co-migration of proteins with similar molecular weights and pIs [15]. A co-migrating protein with similar pI and MW as S28 could account for this difference.

The proteomic analysis of three F2 vector genotypes identified that both cyclophilin isoforms (S28 and S29) were present. Unlike the parent, the relative amount of S28 was greater than S29 [17] so deamination is unlikely to be responsible for the high amount of S28, but a co-migrating protein could account for this difference. Genomic analysis of two of these three F2 genotypes (A3 and G11, G8 was lost) indicated that both alleles were present, which would account for the high levels of both cyclophilin isoforms observed on the gels.

Surprisingly, none of the F2s analyzed were homozygous for the vector allele. However, because we only have analyzed 12 of the F2s and only 3 are good vectors, we may have a biased population. The correlation of the presence of the vector allele of cyclophilin with efficient CYDV-RPV transmission was extended to a number of field collected populations of *S* *. graminum* that can be separated into distinct biotypes [35]. Four of the 11 biotypes were efficient vectors of CYDV-RPV and all encoded the vector isoform of cyclophilin, whereas the seven biotypes showing lower vector efficiency only encoded the nonvector isoform. Genotypes lacking the vector isoform of cyclophilin but transmitting CYDV-RPV with lower efficiency express protein isoforms conferring vector competence [15,16]. In these genetic backgrounds, the nonvector isoform of cyclophilin may be sufficient to allow virus transmission. *S* *. graminum* biotypes can be divided into two categories according to the host plant preference: some biotypes are adapted to agronomic crops whereas others colonize wild grass species [46]. Previously, we had reported a correlation between virus transmission phenotype and host adaptation of *S* *. graminum* [47] and that the ability to colonize cultivated crops might have come at the expense of the ability to transmit viruses causing yellow dwarf disease. A role of cyclophilin in host adaptation is unknown, but the cyclophilin isoform may serve as a valuable biomarker to rapidly identify risk factors of *S* *. graminium* populations as virus vectors and pests of agronomic cereal crops [15].

Three of these nonvector F2 genotypes, C2, K2 and K3 were used in the proteomic analysis and only the S28 protein was detected. Similar to the nonvector parent that contained only the nonvector allele, a majority of the F2 genotypes with low transmission efficiencies were homozygous for the nonvector allele. Interestingly, the two nonvector genotypes that were identified as having both cyclophilin alleles (BB1 and K3) were found to have a strong salivary gland barrier and were lacking a hindgut barrier (Table 2) [28]. In these genotypes, virus is transported across the gut tissues similar as in the vector parental genotype. The nonvector parent and the F2 genotypes that were homozygous for the nonvector allele have at least a hindgut barrier to CYDV-RPV transmission (Table 2). Cyclophilin has not yet been localized to any particular aphid tissues, but these results lead to the hypothesis that the S29 isoform of cyclophilin may be involved in the efficient transport of CYDV-RPV across the hindgut of *S* *. graminum* . Although the accessory salivary gland is the site that determines vector specificity for most aphid-BYDV/CYDV combinations [2], the hindgut can act as a virus specific barrier and CYDV-RPV was shown to be a virus whose transport through the aphid is regulated at the hindgut [4].

Both isoforms of *S* *. graminum* cyclophilin B fused to a his tag interacted with CYDV-RPV but did not interact detectably with PLRV (Figure 2). PLRV is a polerovirus related to CYDV-RPV but with different vector specificities. There is one major difference in the circulative pathway PLRV and CYDV-RPV take through the aphid. CYDV-RPV is acquired through the hindgut of the vector *S* *. graminum* [9,48] whereas PLRV is acquire through the midgut of *M* *. persicae* [49]. The tissue tropism in the vector is determined by one of the virus structural proteins [7], but the virus must interact with different aphid proteins in the hindgut and midgut. If cyclophilin functions during hindgut transport of the virus as the genetic correlation data suggests, this would also provide a plausible explanation for the interaction with CYDV-RPV and not PLRV.

The *in vitro* his-tagged cyclophilin interacted with CYDV-RPV but we did not detect this interaction using co-IP from total aphid protein homogenate. Yang et al. (2008) reported an interaction between CYDV-RPV and a single protein of the similar molecular weight and pI as cyclophilin B (S29) using co-IP-DIGE, but this protein was never identified to be cyclophilin using mass spectrometry [17]. The identification of a single protein assumed to be S29 as interacting with CYDV-RPV in the Yang et al. (2008) experiment conflicts with the his-tagging experiment (Figure 2) that showed both S28 and S29 interact with CYDV-RPV. Our co-IP experiment with aphid and CYDV-RPV indicates the potential involvement of a third cyclophilin protein, cyclophilin A. One hypothesis is that the protein spot from the co-IP-DIGE reported by Yang et al. 2008 contained cyclophilin A and not either of the two cyclophilin B isoforms or perhaps a mixture of both. The difference in results could be explained by the differences in techniques used and the detection limits of each analytical platform. Indeed the cyclophilin A identified in complex with CYDV-RPV has a predicted pI and MW of 9.45 and 22.8, respectively, very similar to the cyclophilin B isoforms. These new data on cyclophilin A provide a more parsimonious explanation for the discrepancy in the data from the his-tagging experiment reported here and the co-IP-DIGE observations reported previously [17]. Speculative and yet reasonable explanations for why we did not identify cyclophilin B using the co-IP-LC-MS/MS approach include (a) that cyclophilin A outcompeted cyclophilin B for binding CYDV-RPV in the presence of both cyclophilins, (b) the washing procedure removed cyclophilin B from the complex, or (c) that cyclophilin B was not detectable in the complex matrix of the co-IP due to the incubation time that was used. Incubation times are known to have an impact on protein recovery during co-IP [50].

The cyclophilin B 29 allele and isoform expression is predictive of vectoring capacity in *S* *. graminum* but binding of the cyclophilin proteins to CYDV-RPV is not. The reasons for the difference in transmission efficiency in these aphids when cyclophilin A and B from vector and nonvector aphids interact (directly or in complex) with CYDV-RPV are not clear. Both cyclophilins have a signal peptide that is predicted to be cleaved; however, we cannot exclude the possibility that they have different subcellular localization. The differing amino acid between the cyclophilin B isoforms is located in position 2 of the mature form. The glutamine residue present in the vector isoform is neutral whereas the glutamic acid residue in the nonvector isoform is acidic. This difference in charge may be responsible for changes in the tertiary structure of the protein resulting in different affinities for the virions or with other proteins. In spite of the *in vitro* interactions between cyclophilin A and B and CYDV-RPV, cyclophilin could be involved in virus transmission by another mechanism besides direct interaction with virions. Cyclophilins have been shown in complexes on mammalian cell surfaces despite the lack of domains explaining the association with plasma membranes [32]. Therefore, aphid cyclophilin proteins may be associated with other key plasma membrane proteins that function in virus recognition. In animals, cyclophilin proteins are now widely recognized to play diverse roles in virus–host interactions for vesicular stomatitis virus [51], coronavirus [52], human immunodeficiency virus [53], hepatitis C [54] and vaccina virus [55].

The precise roles of cyclophilin A or B in CYDV-RPV transmission have not been shown directly. However, the combination of genetic and biochemical data for a role in transmission is the strongest to date for any aphid protein being involved in virus transmission. Luteovirus transmission is a polygenic character governed by few major genes and several minor genes acting in an additive manner. Our results point to a role of these cyclophilin proteins in CYDV-RPV transmission, probably during crossing of the hindgut. The vector isoform of cyclophilin B (S29) does not appear to essential for CYDV-RPV transmission but might play an important role in facilitating the process. Continuing work is focused on localizing cyclophilin A and B in specific aphid tissues and providing evidence of an *in vivo* interaction of virus and cyclophilin in vector and nonvector aphid genotypes and aphid species. These results will help us to draw the boundary of how cyclophilin regulates yellow dwarf virus transmission. The results also highlight the importance of forging deeper connections between genomic and proteomic variation underlying complex phenotypes such as virus transmission by insects.

## Supporting Information

Figure S1 Translation of contig from 454 sequencing of Biotype H cDNA head and gut library containing the cyclophilin A gene sequence. Translation of *S* *. graminum* cyclophilin A from a 454-generated cDNA sequence database that was identified in a co-IP reaction using aphid proteins and purified CYDV-RPV.
(PDF)

Figure S2 Sequence alignment of the 5 cyclophilin alleles identified. CV: allele encoding the vector isoform. CNV1-4: alleles encoding the nonvector isoform. Boxes show the nucleotide differences among alleles. * shows the unique nonsynonymous change in position 94. CV and CNV1 represent the alleles identified in the vector and nonvector parent, respectively.
(PDF)

Figure S3 Tandem mass spectra and retention times for two peptides used in label-free quantification of cyclophilin in aphid-virus co-immunoprecipitation reactions. The doubly charged, fully tryptic peptide FFDMTAFGEQLR was selected for tandem MS at retention times 41.3-41.5 min (A). Retention time CV is 0.2% (B). The triple charged, fully tryptic peptide HTGPGILSMANAGANTNGSQFFITTVK was selected for tandem MS at retention time 42.2 (C). Retention time CV is 0.2%.
(PDF)

Dataset S1 Raw and normalized total peak area for two peptides used for label-free quantification of cyclophilin A in co-immunoprecipitation reaction. (PDF)

Dataset S2 Spectral counting peptide data from Table 3 for cyclophilin A. (PDF)

## References

[1] Miller WA, Rasochová L (1997) Barley yellow dwarf viruses. Annu Rev Phytopathol 35: 167-190. doi:10.1146/annurev.phyto.35.1.167. PubMed: 15012520. 15012520 10.1146/annurev.phyto.35.1.167

[2] Gray S, Gildow FE (2003) Luteovirus-aphid interactions. Annu Rev Phytopathol 41: 539-566. doi:10.1146/annurev.phyto.41.012203.105815. PubMed: 12730400. 12730400 10.1146/annurev.phyto.41.012203.105815

[3] Herrbach É (1999) Vector-virus interactions. In: Smith HG Barker H The Luteoviridae. New York. Wallingford: CAB International pp. 85-88.

[4] Gildow FE (1993) Evidence for receptor-mediated endocytosis regulating luteovirus acquisition by aphids. Phytopathology 83: 270-277. doi:10.1094/Phyto-83-270.

[5] Smith HG, Barker H (1999) The Luteoviridae. Wallingford: CABI Publishing p. 297.

[6] Miller WA, Allan G, Robert GW (1999) Luteovirus (Luteoviridae). Encyclopedia Virology Oxf Elsevier: 901-908.

[7] Brault V, Périgon S, Reinbold C, Erdinger M, Scheidecker D et al. (2005) The polerovirus minor capsid protein determines vector specificity and intestinal tropism in the aphid. J Virol 79: 9685-9693. doi:10.1128/JVI.79.15.9685-9693.2005. PubMed: 16014930. 16014930 10.1128/JVI.79.15.9685-9693.2005 PMC1181584

[8] Chay CA, Gunasinge UB, Dinesh-Kumar SP, Miller WA, Gray SM (1996) Aphid transmission and systemic plant infection determinants of barley yellow dwarf luteovirus-PAV are contained in the coat protein readthrough domain and 17-kDa protein, respectively. Virology 219: 57-65. doi:10.1006/viro.1996.0222. PubMed: 8623554. 8623554 10.1006/viro.1996.0222

[9] Gildow F (1999) Luteovirus transmission mechanisms regulating vector specificity. In: Smith HG Barker H The Luteoviridae. New York. Wallingford: CAB International pp. 88-111.

[10] van den Heuvel JF, Verbeek M, Peters D (1993) The relationship between aphid-transmissibility of Potato leafroll virus and surface epitopes of the viral capsid. Phytopathology 83: 1125-1129. doi:10.1094/Phyto-83-1125.

[11] Chavez JD, Cilia M, Weisbrod CR, Ju HJ, Eng JK et al. (2012) Cross-linking measurements of the Potato leafroll virus reveal protein interaction topologies required for virion stability, aphid transmission, and virus-plant interactions. J Proteome Res 11: 2968-2981. doi:10.1021/pr300041t. PubMed: 22390342. 22390342 10.1021/pr300041t PMC3402239

[12] Li C, Cox-Foster D, Gray SM, Gildow F (2001) Vector specificity of barley yellow dwarf virus (BYDV) transmission: identification of potential cellular receptors binding BYDV-MAV in the aphid, Sitobion avenae. Virology 286: 125-133. doi:10.1006/viro.2001.0929. PubMed: 11448166. 11448166 10.1006/viro.2001.0929

[13] Seddas P, Boissinot S, Strub JM, Van Dorsselaer A, Van Regenmortel MH et al. (2004) Rack-1, GAPDH3, and actin: proteins of Myzus persicae potentially involved in the transcytosis of beet western yellows virus particles in the aphid. Virology 325: 399-412. doi:10.1016/j.virol.2004.05.014. PubMed: 15246278. 15246278 10.1016/j.virol.2004.05.014

[14] Wang X, Zhou G (2003) Identification of a protein associated with circulative transmission of Barley yellow dwarf virus from cereal aphids, *Schizaphis graminum* and *Sitobion avenae* . Chin Sci Bull 48: 2083-2087. doi:10.1360/03wc0153.

[15] Cilia M, Howe K, Fish T, Smith D, Mahoney J et al. (2011) Biomarker discovery from the top down: Protein biomarkers for efficient virus transmission by insects (Homoptera: Aphididae) discovered by coupling genetics and 2-D DIGE. Proteomics 11: 2440-2458. doi:10.1002/pmic.201000519. PubMed: 21648087. 21648087 10.1002/pmic.201000519

[16] Cilia M, Tamborindeguy C, Fish T, Howe K, Thannhauser TW et al. (2011) Genetics coupled to quantitative intact proteomics links heritable aphid and endosymbiont protein expression to circulative polerovirus transmission. J Virol 85: 2148-2166. doi:10.1128/JVI.01504-10. PubMed: 21159868. 21159868 10.1128/JVI.01504-10 PMC3067806

[17] Yang X, Thannhauser TW, Burrows M, Cox-Foster D, Gildow FE et al. (2008) Coupling genetics and proteomics to identify aphid proteins associated with vector-specific transmission of polerovirus (luteoviridae). J Virol 82: 291-299. doi:10.1128/JVI.01736-07. PubMed: 17959668. 17959668 10.1128/JVI.01736-07 PMC2224398

[18] Choi DS, Young H, McMahon T, Wang D, Messing RO (2003) The mouse RACK1 gene is regulated by nuclear factor-kappa B and contributes to cell survival. Mol Pharmacol 64: 1541-1548. doi:10.1124/mol.64.6.1541. PubMed: 14645685. 14645685 10.1124/mol.64.6.1541

[19] Tisdale EJ (2002) Glyceraldehyde-3-phosphate dehydrogenase is phosphorylated by protein kinase Ciota /lambda and plays a role in microtubule dynamics in the early secretory pathway. J Biol Chem 277: 3334-3341. doi:10.1074/jbc.M109744200. PubMed: 11724794. 11724794 10.1074/jbc.M109744200

[20] Apodaca G (2001) Endocytic traffic in polarized epithelial cells: role of the actin and microtubule cytoskeleton. Traffic 2: 149-159. doi:10.1034/j.1600-0854.2001.020301.x. PubMed: 11260520. 11260520 10.1034/j.1600-0854.2001.020301.x

[21] Ploubidou A, Way M (2001) Viral transport and the cytoskeleton. Curr Opin Cell Biol 13: 97-105. doi:10.1016/S0955-0674(00)00180-0. PubMed: 11163140. 11163140 10.1016/S0955-0674(00)00180-0 PMC7125730

[22] Jaubert-Possamai S, Rispe C, Tanguy S, Gordon K, Walsh T et al. (2010) Expansion of the miRNA pathway in the hemipteran insect Acyrthosiphon pisum. Mol Biol Evol 27: 979-987. doi:10.1093/molbev/msp256. PubMed: 20179251. 20179251 10.1093/molbev/msp256 PMC2857804

[23] Mutti NS, Park Y, Reese JC, Reeck GR (2006) RNAi knockdown of a salivary transcript leading to lethality in the pea aphid, Acyrthosiphon pisum. J Insect Sci 6: 1-7. doi:10.1673/031.006.3801. 10.1673/031.006.3801 PMC2990334 20233093

[24] Pitino M, Coleman AD, Maffei ME, Ridout CJ, Hogenhout SA (2011) Silencing of aphid genes by dsRNA feeding from plants. PLOS ONE 6: e25709. doi:10.1371/journal.pone.0025709. PubMed: 21998682. 21998682 10.1371/journal.pone.0025709 PMC3187792

[25] IAGC (2010) Genome sequence of the pea aphid Acyrthosiphon pisum. PLOS Biol 8: e1000313. 20186266 10.1371/journal.pbio.1000313 PMC2826372

[26] Srinivasan DG, Brisson JA (2012) Aphids: a model for polyphenism and epigenetics. Genet Res Int, 2012: 2012: 431531. PubMed: 22567389 10.1155/2012/431531 PMC3335499 22567389

[27] Simon JC, Pfrender ME, Tollrian R, Tagu D, Colbourne JK (2011) Genomics of environmentally induced phenotypes in 2 extremely plastic arthropods. J Hered 102: 512-525. doi:10.1093/jhered/esr020. PubMed: 21525179. 21525179 10.1093/jhered/esr020 PMC3156564

[28] Burrows ME, Caillaud MC, Smith DM, Benson EC, Gildow FE et al. (2006) Genetic regulation of polerovirus and luteovirus transmission in the aphid Schizaphis graminum. Phytopathology 96: 828-837. doi:10.1094/PHYTO-96-0828. PubMed: 18943747. 18943747 10.1094/PHYTO-96-0828

[29] Burrows ME, Caillaud MC, Smith DM, Gray SM (2007) Biometrical genetic analysis of luteovirus transmission in the aphid Schizaphis graminum. Heredity 98: 106-113. doi:10.1038/sj.hdy.6800909. PubMed: 17021612. 17021612 10.1038/sj.hdy.6800909

[30] Takahashi N, Hayano T, Suzuki M (1989) Peptidyl-prolyl cis-trans isomerase is the cyclosporin A-binding protein cyclophilin. Nature 337: 473-475. doi:10.1038/337473a0. PubMed: 2644542. 2644542 10.1038/337473a0

[31] Hasel KW, Glass JR, Godbout M, Sutcliffe JG (1991) An endoplasmic reticulum-specific cyclophilin. Mol Cell Biol 11: 3484-3491. PubMed: 1710767. 1710767 10.1128/mcb.11.7.3484-3491.1991 PMC361082

[32] Price ER, Jin M, Lim D, Pati S, Walsh CT et al. (1994) Cyclophilin B trafficking through the secretory pathway is altered by binding of cyclosporin A. Proc Natl Acad Sci U S A 91: 3931-3935. doi:10.1073/pnas.91.9.3931. PubMed: 7909608. 7909608 10.1073/pnas.91.9.3931 PMC43696

[33] Pemberton TJ, Kay JE (2005) Identification and Comparative Analysis of the Peptidyl-Prolyl cis/trans Isomerase Repertoires of H. sapiens, D. melanogaster, C. elegans, S. cerevisiae and Sz. pombe. Comp Funct Genomics 6: 277-300. doi:10.1002/cfg.482. PubMed: 18629211. 18629211 10.1002/cfg.482 PMC2447506

[34] Katsar C, Gray S (1999) Rearing aphids to use in virus-vectors studies. In: Maramorosch K Mahmood F Maintenance of human, animal, and plant pathogen vectors. Enfield: Science Publishing House Publishers, Inc. pp. 183-195.

[35] Porter DR, Burd JD, Shufran KA, Webster JA, Teetes GL (1997) Greenbug (Homoptera: Aphididae) biotypes: Selected by resistant cultivars or preadapted opportunists? J Econ Entomol 90: 1055-1065.

[36] Lee L, Kaplan IB, Ripoll DR, Liang D, Palukaitis P et al. (2005) A Surface Loop of the Potato Leafroll Virus Coat Protein Is Involved in Virion Assembly, Systemic Movement, and Aphid Transmission. J Virol 79: 1207-1214. doi:10.1128/JVI.79.2.1207-1214.2005. PubMed: 15613347. 15613347 10.1128/JVI.79.2.1207-1214.2005 PMC538549

[37] Gasteiger E, Gattiker A, Hoogland C, Ivanyi I, Appel RD et al. (2003) ExPASy: the proteomics server for in-depth protein knowledge and analysis. Nucleic Acids Res 31: 3784-3788. doi:10.1093/nar/gkg563. PubMed: 12824418. 12824418 10.1093/nar/gkg563 PMC168970

[38] Cristea IM, Chait BT (2011) Conjugation of magnetic beads for immunopurification of protein complexes. Cold Spring Harb Protoc. 2011/05/04 ed 10.1101/pdb.prot5610 PMC6666400 21536766

[39] Chambers MC, Maclean B, Burke R, Amodei D, Ruderman DL et al. (2012) A cross-platform toolkit for mass spectrometry and proteomics. Nat Biotechnol 30: 918-920. doi:10.1038/nbt.2377. PubMed: 23051804. 23051804 10.1038/nbt.2377 PMC3471674

[40] Keller A, Nesvizhskii AI, Kolker E, Aebersold R (2002) Empirical statistical model to estimate the accuracy of peptide identifications made by MS/MS and database search. Anal Chem 74: 5383-5392. doi:10.1021/ac025747h. PubMed: 12403597. 12403597 10.1021/ac025747h

[41] MacLean B, Tomazela DM, Shulman N, Chambers M, Finney GL et al. (2010) Skyline: an open source document editor for creating and analyzing targeted proteomics experiments. Bioinformatics 26: 966-968. doi:10.1093/bioinformatics/btq054. PubMed: 20147306. 20147306 10.1093/bioinformatics/btq054 PMC2844992

[42] Schilling B, Rardin MJ, Maclean BX, Zawadzka AM, Frewen BE et al. (2012) Platform independent and label-free quantitation of proteomic data using MS1 extracted ion chromatograms in skyline. Application to protein acetylation and phosphorylation. Mol Cell Proteomics. 10.1074/mcp.M112.017707 PMC3418851 22454539

[43] Burd JD, Porter DR (2006) Biotypic diversity in greenbug (Hemiptera: Aphididae): characterizing new virulence and host associations. J Econ Entomol 99: 959-965. doi:10.1603/0022-0493-99.3.959. PubMed: 16813337. 16813337 10.1603/0022-0493-99.3.959

[44] Power AG, Gray SM (1995) Aphid transmission of barley yellow dwarf viruses: interactions between viruses, vectors, and host plants. In: D’Arcy CJ Burnett PA Barley Yellow Dwarf: 40 Years of Progress. St. Paul: APS Press pp. 259-289.

[45] Robinson AB (1974) Evolution and the Distribution of Glutaminyl and Asparaginyl Residues in Proteins. Proc Natl Acad Sci U S A 71: 885-888. doi:10.1073/pnas.71.3.885. PubMed: 4522799. 4522799 10.1073/pnas.71.3.885 PMC388120

[46] Shufran KA, Burd JD, Anstead JA, Lushai G (2000) Mitochondrial DNA sequence divergence among greenbug (Homoptera: Aphididae) biotypes: evidence for host-adapted races. Insect Mol Biol 9: 179-184. doi:10.1046/j.1365-2583.2000.00177.x. PubMed: 10762425. 10762425 10.1046/j.1365-2583.2000.00177.x

[47] Gray SM, Smith DM, Barbierri L, Burd J (2002) Virus Transmission Phenotype Is Correlated with Host Adaptation Among Genetically Diverse Populations of the Aphid Schizaphis graminum. Phytopathology 92: 970-975. doi:10.1094/PHYTO.2002.92.9.970. PubMed: 18944022. 18944022 10.1094/PHYTO.2002.92.9.970

[48] Cilia M, Peter KA, Bereman MS, Howe K, Fish T et al. (2012) Discovery and targeted LC-MS/MS of purified polerovirus reveals differences in the virus-host interactome associated with altered aphid transmission. PLOS ONE 7: e48177. doi:10.1371/journal.pone.0048177. PubMed: 23118947. 23118947 10.1371/journal.pone.0048177 PMC3484124

[49] Garret A, Kerlan C, Thomas D (1993) The intestine is a site of passage for potato leafroll virus from the gut lumen into the haemocoel in the aphid vector, Myzus persicae Sulz. Arch Virol 131: 377-392. 8347080 10.1007/BF01378639

[50] Cristea IM, Williams R, Chait BT, Rout MP (2005) Fluorescent proteins as proteomic probes. Mol Cell Proteomics 4: 1933-1941. doi:10.1074/mcp.M500227-MCP200. PubMed: 16155292. 16155292 10.1074/mcp.M500227-MCP200

[51] Bose S, Mathur M, Bates P, Joshi N, Banerjee AK (2003) Requirement for cyclophilin A for the replication of vesicular stomatitis virus New Jersey serotype. J Gen Virol 84: 1687-1699. doi:10.1099/vir.0.19074-0. PubMed: 12810862. 12810862 10.1099/vir.0.19074-0

[52] Pfefferle S, Schöpf J, Kögl M, Friedel CC, Müller MA et al. (2011) The SARS-coronavirus-host interactome: identification of cyclophilins as target for pan-coronavirus inhibitors. PLOS Pathog 7: e1002331 PubMed: 22046132. 22046132 10.1371/journal.ppat.1002331 PMC3203193

[53] Takemura T, Kawamata M, Urabe M, Murakami T (2013) Cyclophilin A-dependent restriction to capsid N121K mutant Human Immunodeficiency Virus Type 1 in a broad range of cell lines. J Virol. 10.1128/JVI.01319-12 PMC3624240 23325683

[54] Hanoulle X, Badillo A, Wieruszeski JM, Verdegem D, Landrieu I et al. (2009) Hepatitis C virus NS5A protein is a substrate for the peptidyl-prolyl cis/trans isomerase activity of cyclophilins A and B. J Biol Chem 284: 13589-13601. doi:10.1074/jbc.M809244200. PubMed: 19297321. 19297321 10.1074/jbc.M809244200 PMC2679460

[55] Castro AP, Carvalho TM, Moussatché N, Damaso CR (2003) Redistribution of cyclophilin A to viral factories during vaccinia virus infection and its incorporation into mature particles. J Virol 77: 9052-9068. doi:10.1128/JVI.77.16.9052-9068.2003. PubMed: 12885921. 12885921 10.1128/JVI.77.16.9052-9068.2003 PMC167230

