# Supplementary material for: Genomic and Proteomic Analysis of Schizaphis graminum Reveals Cyclophilin Proteins Are Involved in the Transmission of Cereal Yellow Dwarf Virus
Source: PLoS One. 2013 Aug 9;8(8):e71620. doi: 10.1371/journal.pone.0071620 (PMC3739738; doi:10.1371/journal.pone.0071620)
Supplement: Figure S1 — Translation of S . graminum cyclophilin A from a 454-generated cDNA sequence database that was identified in a co-IP reaction using aphid proteins and purified CYDV-RPV. (PDF) [file pone.0071620.s001.pdf]

>Biotype H Cyclophilin Contig Partial
23879 (translated in frame 1)
TMASTTFRLF TRRQFLYCST PNHSYTGGLA SFLLYSTKSK PSSSKMSLPK
VFFDMTADGE QLGRITIELR SDVVPKTAEN FRALCTGEKG FGYKGSIFHR
VIPNFMCQGG DFTNHNGTGG KSIYGNKFED ENFTLKHTGP GILSMANAGA
NTNGSQFFIT TVKTSWLDTK HVVFGAIVDG MDVVKKIESY GTQSGKTTKK
ITVANCGQLS
