# Supplementary material for: Genomic and Proteomic Analysis of Schizaphis graminum Reveals Cyclophilin Proteins Are Involved in the Transmission of Cereal Yellow Dwarf Virus
Source: PLoS One. 2013 Aug 9;8(8):e71620. doi: 10.1371/journal.pone.0071620 (PMC3739738; doi:10.1371/journal.pone.0071620)
Supplement: Figure S2 — CV: allele encoding the vector isoform. CNV1-4: alleles encoding the nonvector isoform. Boxes show the nucleotide differences among alleles. * shows the unique nonsynonymous change in position 94. CV and CNV1 represent the alleles identified in the vector and nonvector parent, respectively. (PDF) [file pone.0071620.s002.pdf]

Figure S2

```
              ....|....| ....|....| ....|....| ....|....| ....|....|
                      10         20         30         40         50
CV            ATGATATCTA CTTATAAAAT CATGACGTTA TTGAAATGGG CTATATTGCC
CNV1          ATGATATCTA CTTATAAAAT CATGACGTTA TTGAAATGGG CTATATTGCC
CNV2          ATGATATCTA CTTATAAAAT CATGACGTTA TTGAAATGGG CTATATTGCC
CNV3          ATGATATCTA CTTATAAAAT CATGACGTTA TTGAAATGGG CTATATTGCC
CNV4          ATGATATCTA CTTATAAAAT CATGACGTTA TTGAAATGGG CTATATTGCC


              ....|....| ....|....| ....|....| ....|....| ....|....|
                      60         70         80         90   *    100
CV            GTTAACGGTA TTCACGATTT TAGTGGTGTA TAGCCAAGCA TTACAAGACC
CNV1          GTTAACGGTA TTCACGATTT TAGTGGTGTA TAGCCAAGCA TTAGAAGACC
CNV2          GTTAACGGTA TTCACGATTT TAGTGGTGTA TAGCCAAGCA TTAGAAGACC
CNV3          GTTAACGGTA TTCACGATTT TAGTGGTGTA TAGCCAAGCA TTAGAAGACC
CNV4          GTTAACGGTA TTCACGATTT TAGTGGTGTA TAGCCAAGCA TTAGAAGACC


              ....|....| ....|....| ....|....| ....|....| ....|....|
                     110        120        130        140        150
CV            CACAGAAGAA AGGACCTAAG GTCACTGACA TTGTATGGTT CGACATCAAA
CNV1          CACAGAAGAA AGGACCTAAG GTCACTGACA TTGTATGGTT CGACATCAAA
CNV2          CACAGAAGAA AGGACCTAAG GTCACTGACA TTGTATGGTT CGACATCAAA
CNV3          CACAGAAGAA AGGACCTAAG GTCACTGACA TTGTATGGTT CGACATCAAA
CNV4          CACAGAAGAA AGGACCTAAG GTCACTGACA TTGTATGGTT CGACATCAAA


              ....|....| ....|....| ....|....| ....|....| ....|....|
                     160        170        180        190        200
CV            ATTGGTTCAA GTGAACCTCA ACGTGTGGAA ATCGGAGTAT TTGGTGCAAC
CNV1          ATTGGTTCAA GTGAACCTCA ACGTGTGGAA ATCGGAGTAT TTGGTGCAAC
CNV2          ATTGGTTCAA GTGAACCTCA ACGTGTGGAA ATCGGAGTAT TTGGTGCAAC
CNV3          ATTGGTTCAA GTGAACCTCA ACGTGTGGAA ATCGGAGTAT TTGGTGCAAC
CNV4          ATTGGTTCAA GTGAACCTCA ACGTGTGGAA ATCGGAGTAT TTGGTGCAAC


              ....|....| ....|....| ....|....| ....|....| ....|....|
                     210        220        230        240        250
CV            TGTACCTAAA ACTGCAAAGA ATTTTATTGA ACTAGCAAAA AAACCAGAAG
CNV1          TGTACCTAAA ACTGCAAAGA ATTTTATTGA ACTAGCAAAA AAACCAGAAG
CNV2          TGTACCTAAA ACTGCAAAGA ATTTTATTGA ACTAGCAAAA AAACCAGAAG
CNV3          TGTACCTAAA ACTGCAAAGA ATTTTATTGA ACTAGCAAAA AAACCAGAAG
CNV4          TGTGCCTAAA ACTGCAAAGA ATTTTATTGA ACTAGCAAAA AAACCAGAAG


              ....|....| ....|....| ....|....| ....|....| ....|....|
                     260        270        280        290        300
CV            GTGAAGGATA CAAGGGCAGT AAATTCCATA GAGTTATCAA AGACTTCATG
CNV1          GTGAAGGATA CAAGGGCAGT AAATTCCATA GAGTTATCAA AGACTTCATG
CNV2          GTGAAGGATA CAAGGGCAGT AAATTCCATA GAGTTATCAA AGACTTCATG
CNV3          GTGAAGGATA CAAGGGTAGT AAATTCCATA GAGTTATCAA AGACTTCATG
CNV4          GTGAAGGATA CAAGGGCAGT AAATTCCATA GAGTTATCAA AGACTTCATG


              ....|....| ....|....| ....|....| ....|....| ....|....|
                     310        320        330        340        350
CV            ATCCAAGGAG GTGATTTTAC AAAAGGTGAC GGCACAGGTG GTCGCAGTAT
CNV1          ATCCAAGGAG GTGATTTTAC AAAAGGTGAC GGCACAGGTG GTCGCAGTAT
CNV2          ATCCAAGGAG GTGATTTTAC AAAAGGTGAC GGCACAGGTG GTCGCAGTAT
CNV3          ATCCAAGGAG GTGATTTTAC AAAAGGTGAC GGCACAGGTG GTCGCAGTAT
CNV4          ATCCAAGGAG GTGATTTTAC AAAAGGTGAC GGCACAGGTG GTCGCAGTAT


              ....|....| ....|....| ....|....| ....|....| ....|....|
                     360        370        380        390        400
CV            TTTTGGTGAA AAGTTTGCAG ATGAAAACTT CAAGCTGAAA CATTATGGTG
CNV1          TTTTGGTGAA AAGTTTGCAG ATGAAAACTT CAAGCTGAAA CATTATGGTG
CNV2          TTTTGGTGAA AAGTTTGCAG ATGAAAACTT CAAGCTGAAA CATTATGGCG
CNV3          TTTTGGTGAA AAGTTTGCAG ATGAAAACTT CAAGCTGAAA CATTATGGTG
CNV4          TTTTGGTGAA AAGTTTGCAG ATGAAAACTT CAAGCTGAAA CATTATGGTG


              ....|....| ....|....| ....|....| ....|....| ....|....|
                     410        420        430        440        450
CV            CTGGATGGTT GTCTATGGCC AATGCAGGAA AAGATACCAA TGGATCTCAA
CNV1          CTGGTTGGTT GTCTATGGCC AATGCAGGAA AAGATACCAA TGGATCTCAA
CNV2          CTGGTTGGTT GTCTATGGCC AATGCAGGAA AAGATACCAA TGGATCTCAA
CNV3          CTGGTTGGTT GTCTATGGCC AATGCAGGAA AAGATACCAA TGGATCTCAA
CNV4          CTGGTTGGTT GTCTATGGCC AATGCAGGAA AAGATACCAA TGGATCTCAA


              ....|....| ....|....| ....|....| ....|....| ....|....|
                     460        470        480        490        500
CV            TTTTTCATTA CCACTAAACA AACTTCCTGG TTGGACGGTC GCCATGTTGT
CNV1          TTTTTCATTA CCACTAAACA AACTTCCTGG TTGGACGGTC GCCATGTTGT
CNV2          TTTTTCATTA CCACTAAACA AACTTCCTGG TTGGACGGTC GCCATGTTGT
CNV3          TTTTTCATTA CCACTAAACA AACTTCCTGG TTGGACGGTC GCCATGTTGT
CNV4          TTTTTCATTA CCACTAAACA AACTTCCTGG TTGGACGGTC GCCATGTTGT


              ....|....| ....|....| ....|....| ....|....| ....|....|
                     510        520        530        540        550
CV            GTTTGGAAAA ATAATCAAAG GAATGAAAAC AATTAGAGCA GCTGAATCAG
CNV1          GTTTGGAAAA ATAATCAAAG GAATGAAAAC AATTAGAGCA GCTGAATCAG
CNV2          GTTTGGAAAA ATAATCAAAG GAATGAAAAC AATTAGAGCA GCTGAATCAG
CNV3          GTTTGGAAAA ATAATCAAAG GAATGAAAAC AATTAGAGCA GCTGAATCAG
CNV4          GTTTGGAAAA ATAATCAAAG GAATGAAAAC AATTAGAGCA GCTGAATCAG


              ....|....| ....|....| ....|....| ....|....| ....|....|
                     560        570        580        590        600
CV            CAGAAACTGA TTCCAGAGAC AAACCAGTTG AAAATATAGT AATTATCGAT
CNV1          CAGAAACTGA TTCCAGAGAC AAACCAGTTG AAAATATAGT AATTATCGAT
CNV2          CAGAAACTGA TTCCAGAGAC AAACCAGTTG AAAATATAGT AATTATCGAT
CNV3          CAGAAACTGA TTCCAGAGAC AAACCAGTTG AAAATATAGT AATTATCGAT
CNV4          CAGAAACTGA TTCCAGAGAC AAACCAGTTG AAAATATAGT AATTATCGAT


              ....|....| ....|....| ....|....| ....|....| ....|....|
                     610        620        630        640        650
CV            AGTGGCCACA CCGTCATTTC TGAGCCATAC CCAGTCAGCA AAACTGATGC
CNV1          AGTGGCCACA CCGTCATTTC TGAGCCATAC CCAGTCAGCA AAACTGATGC
CNV2          AGTGGCCACA CCGTCATTTC TGAGCCATAC CCAGTCAGCA AAACTGATGC
CNV3          AGTGGCCACA CCGTGATTTC TGAGCCATAT CCAGTCAGCA AAACTGATGC
CNV4          AGTGGCCACA CCGTCATTTC TGAGCCATAC CCAGTCAGCA AAACTGATGC


              ....|....|
        660
CV            TACCGAATAA
CNV1          TACCGAATAA
CNV2          TACCGAATAA
CNV3          TACCGAATAA
CNV4          TACCGAATAA
```
