# Supplementary figures and images for: Genomic and Proteomic Analysis of Schizaphis graminum Reveals Cyclophilin Proteins Are Involved in the Transmission of Cereal Yellow Dwarf Virus
Source: PLoS One. 2013 Aug 9;8(8):e71620. doi: 10.1371/journal.pone.0071620 (PMC3739738; doi:10.1371/journal.pone.0071620)

A

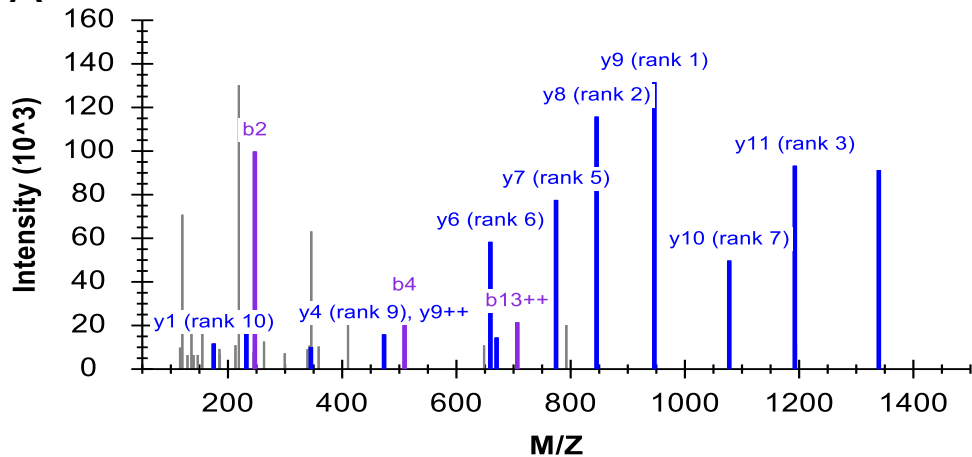

B

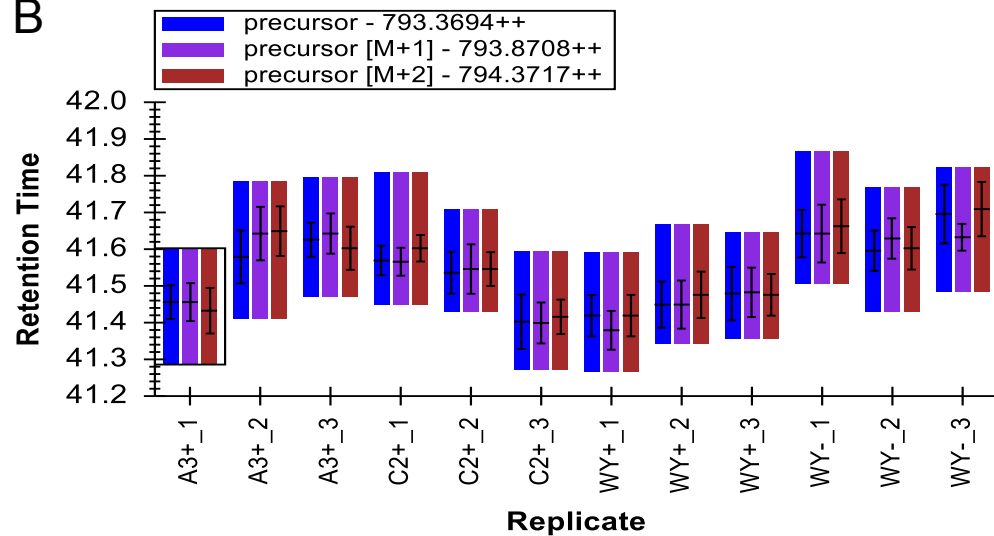

C

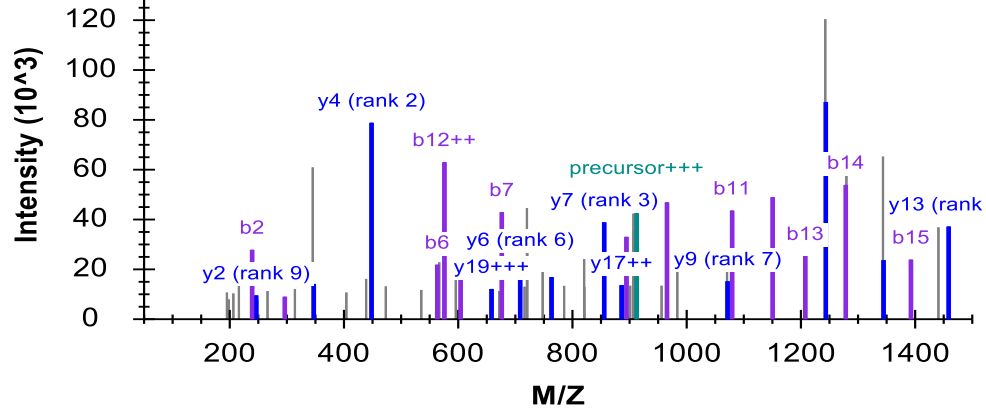

D

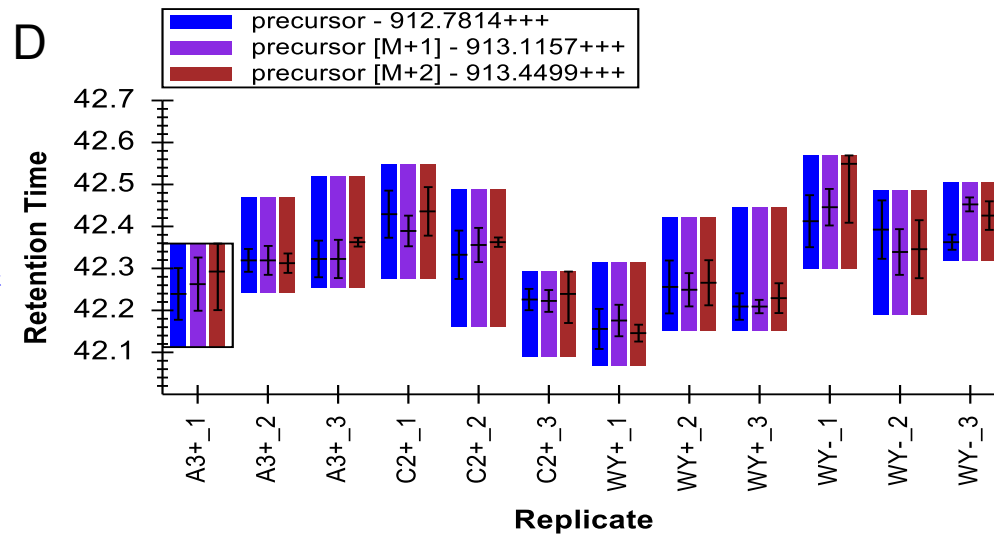

Supplement: Figure S3 — The doubly charged, fully tryptic peptide FFDMTAFGEQLR was selected for tandem MS at retention times 41.3-41.5 min (A). Retention time CV is 0.2% (B). The triple charged, fully tryptic peptide HTGPGILSMANAGANTNGSQFFITTVK was selected for tandem MS at retention time 42.2 (C). Retention time CV is 0.2%. (PDF) [file pone.0071620.s003.pdf]
