# Supplementary material for: Genomic and Proteomic Analysis of Schizaphis graminum Reveals Cyclophilin Proteins Are Involved in the Transmission of Cereal Yellow Dwarf Virus
Source: PLoS One. 2013 Aug 9;8(8):e71620. doi: 10.1371/journal.pone.0071620 (PMC3739738; doi:10.1371/journal.pone.0071620)
Supplement: Dataset S1 — (PDF) [file pone.0071620.s004.pdf]

ProteinName	PeptideSequence	RetentionTime	IsotopeDotPro	ReplicateNar	TotalArea	Normalization Factor	Normalized Peak Area
cyclophilin	VFFDMTADGEQLGR	41.46	0.9983	A3+_1	60167544	0.868936885	52281798.25
cyclophilin	VFFDMTADGEQLGR	41.58	0.9926	A3+_2	61628164	0.937586276	57781720.8
cyclophilin	VFFDMTADGEQLGR	41.63	0.9904	A3+_3	61780072	0.636602686	39329359.8
cyclophilin	VFFDMTADGEQLGR	41.57	0.9872	C2+_1	63425384	0.817058442	51822245.43
cyclophilin	VFFDMTADGEQLGR	41.54	0.9893	C2+_2	66884840	0.694300892	46438204.09
cyclophilin	VFFDMTADGEQLGR	41.4	0.9892	C2+_3	66698792	0.585744595	39068456.9
cyclophilin	VFFDMTADGEQLGR	41.42	0.9961	WY+_1	75452544	0.460637397	34756263.46
cyclophilin	VFFDMTADGEQLGR	41.45	0.9855	WY+_2	70663840	0.380176196	26864709.92
cyclophilin	VFFDMTADGEQLGR	41.48	0.9883	WY+_3	63322448	0.277115949	17547660.26
cyclophilin	VFFDMTADGEQLGR	41.64	0.983	WY-_1	46026608	0.261508297	12036339.88
cyclophilin	VFFDMTADGEQLGR	41.6	0.9783	WY-_2	45677332	0.150213021	6861330.041
cyclophilin	VFFDMTADGEQLGR	41.7	0.9755	WY-_3	38009964	0.098218719	3733289.972
cyclophilin	HTGPGILSMANAGANTI	42.24	0.8396	A3+_1	15400259	0.868936885	13381853.08
cyclophilin	HTGPGILSMANAGANTI	42.32	0.8445	A3+_2	13619700	0.937586276	12769643.81
cyclophilin	HTGPGILSMANAGANTI	42.32	0.8348	A3+_3	13548350	0.636602686	8624916.007
cyclophilin	HTGPGILSMANAGANTI	42.43	0.8856	C2+_1	18961620	0.817058442	15492751.69
cyclophilin	HTGPGILSMANAGANTI	42.33	0.858	C2+_2	22502512	0.694300892	15623514.16
cyclophilin	HTGPGILSMANAGANTI	42.23	0.8753	C2+_3	19737290	0.585744595	11561010.93
cyclophilin	HTGPGILSMANAGANTI	42.16	0.9046	WY+_1	19321264	0.460637397	8900096.754
cyclophilin	HTGPGILSMANAGANTI	42.26	0.8512	WY+_2	16928256	0.380176196	6435719.979
cyclophilin	HTGPGILSMANAGANTI	42.21	0.85	WY+_3	16313295	0.277115949	4520674.223
cyclophilin	HTGPGILSMANAGANTI	42.41	0.8089	WY-_1	6152966	0.261508297	1609051.661
cyclophilin	HTGPGILSMANAGANTI	42.39	0.7304	WY-_2	7928268	0.150213021	1190929.089
cyclophilin	HTGPGILSMANAGANTI	42.36	0.7989	WY-_3	4099046	0.098218719	402603.0471
