# Supplementary material for: Genomic and Proteomic Analysis of Schizaphis graminum Reveals Cyclophilin Proteins Are Involved in the Transmission of Cereal Yellow Dwarf Virus
Source: PLoS One. 2013 Aug 9;8(8):e71620. doi: 10.1371/journal.pone.0071620 (PMC3739738; doi:10.1371/journal.pone.0071620)
Supplement: Dataset S2 — (PDF) [file pone.0071620.s005.pdf]

Cyclophilin A Peptides in WY-10A + CYDV-RPV

Sequence	Prob	Mascot Ion score	Mascot Identity score	Modifications	Observed	Actual Mass	Charge	Delta AMU	Delta PPM	Retention Time	TIC
(-)HTGPGILSMAnAGANTNGSqFFITTVK(-)	95%	27.78	38.50033	Deamidation (+1), Deamidation (+1)	912.79	2,735.35	3	0.02547	9.308	2,527.75	645612
(-)HTGPGILSmANAGANTnGSQFFITTVK(-)	95%	55.43	38.287888	Oxidation (+16), Deamidation (+1)	917.78	2,750.32	3	-0.01543	-5.608	2,359.09	1875350
(-)TSWLDTK(-)	64%	20.14	43.905643		425.7181	849.4216	2	-0.001736	-2.041	1,885.01	3813160
(-)VFFDMTADGEQLGR(-)	95%	61.19	41.564884		793.37	1,584.73	2	0.0009781	0.6168	2,478.46	1552970
(-)VFFDmTADGEQLGR(-)	95%	92.77	41.404766	Oxidation (+16)	801.3665	1,600.72	2	-0.001009	-0.6299	2,233.40	1735810
(-)HTGPGILSmANAGANTNGSqFFITTVK(-)	69%	20.75	38.287888	Oxidation (+16), Deamidation (+1)	917.78	2,750.32	3	-0.01543	-5.608	2,356.14	713233
(-)HTGPGILSmAnAGAnTnGSQFFITTVK(-)	95%	43.67	38.25556	Oxidation (+16), Deamidation (+1), Deamidation (+1), Deamidation (+1)	918.46	2,752.36	3	0.05654	20.53	2,361.11	2948960
(-)HTGPGILSMANAGANTnGSqFFITTVK(-)	95%	48.83	38.50033	Deamidation (+1), Deamidation (+1)	912.79	2,735.35	3	0.02547	9.308	2,532.09	2001440
(-)HVVFGAIVDGMDVVK(-)	82%	20.79	41.58875		529.2848	1,584.83	3	-0.001031	-0.6501	2,376.83	2864390
(-)VFFDMTADGEqLGR(-)	77%	29.58	41.58875	Deamidation (+1)	793.87	1,585.73	2	0.01696	10.69	2,484.56	1700880
(-)HTGPGILSmAnAGAnTnGSQFFITTVK(-)	95%	32.54	38.26269	Oxidation (+16), Deamidation (+1), Deamidation (+1), Deamidation (+1)	918.45	2,752.33	3	0.02654	9.639	2,355.99	1501800
(-)HTGPGILSMANAGANTNGSqFFITTVK(-)	95%	35.95	38.501556	Deamidation (+1)	912.4541	2,734.34	3	0.0019	0.6946	2,533.83	1052200
(-)HTGPGILSmANAGANTnGSQFFITTVK(-)	95%	79.43	38.287888	Oxidation (+16), Deamidation (+1)	917.78	2,750.32	3	-0.01543	-5.608	2,358.48	2513270
(-)TSWLDTK(-)	62%	21.51	44.02657		425.7189	849.4233	2	-7.79E-05	-0.09165	1,893.63	4262090
(-)VFFDmTADGEqLGR(-)	91%	38.21	41.476143	Oxidation (+16), Deamidation (+1)	801.87	1,601.73	2	0.02205	13.76	2,240.35	2444640
(-)VFFDMTADGEQLGR(-)	95%	53.1	41.653038		793.3746	1,584.73	2	0.01016	6.406	2,482.54	2090840

Cyclophilin A Peptides in A3 + CYDV-RPV

Sequence	Prob	Mascot Ion score	Mascot Identity score	Modifications	Observed	Actual Mass	Charge	Delta AMU	Delta PPM	Retention Time	TIC
(-)HVVFGAIVDGMDVVK(-)	79%	27.01	41.690273		529.28	1,584.82	3	-0.0154	-9.71	2,370.15	2921050
(-)HTGPGILSMANAGANTnGSQFFITTVK(-)	83%	22.68	38.459038	Deamidation (+1)	912.46	2,734.36	3	0.01949	7.124	2,540.69	610166
(-)HTGPGILSmANAGANTnGSqFFITTVK(-)	89%	24.41	38.219063	Oxidation (+16), Deamidation (+1), Deamidation (+1)	918.12	2,751.34	3	0.02056	7.468	2,362.20	402686
(-)HTGPGILSmANAGANTNGSqFFITTVK(-)	95%	29.03	38.287888	Oxidation (+16), Deamidation (+1)	917.78	2,750.32	3	-0.01543	-5.608	2,362.81	554338
(-)HVVFGAIVDGMDVVK(-)	95%	33.13	41.4192		529.29	1,584.85	3	0.0146	9.208	2,387.09	3703190
(-)KITVANcGqLS(-)	88%	37.49	42.631386	MMTS (+46), Deamidation (+1)	590.8	1,179.59	2	0.02254	19.09	2,081.77	2335780
(-)VFFDmTADGEQLGR(-)	95%	70.32	41.48078	Oxidation (+16)	801.37	1,600.73	2	0.006063	3.785	2,247.65	1183890
(-)HTGPGILSMANAGAnTnGSqFFITTVK(-)	88%	24.94	38.464607	Deamidation (+1), Deamidation (+1), Deamidation (+1)	913.12	2,736.34	3	0.03145	11.49	2,537.65	714449
(-)HTGPGILSmANAGAnTnGSQFFITTVK(-)	95%	41.75	38.219063	Oxidation (+16), Deamidation (+1), Deamidation (+1)	918.12	2,751.34	3	0.02056	7.468	2,360.27	1466190
(-)VFFDMTADGEqLGR(-)	59%	23.34	41.58875	Deamidation (+1)	793.87	1,585.73	2	0.01696	10.69	2,488.13	848372
(-)VFFDMTADGEQLGR(-)	92%	27.17	41.564884		793.37	1,584.73	2	0.0009781	0.6168	2,486.35	361384
(-)VFFDmTADGEQLGR(-)	95%	62.81	41.48078	Oxidation (+16)	801.37	1,600.73	2	0.006063	3.785	2,243.06	1013440

Cyclophilin A Peptides in C2 + CYDV-RPV

Sequence	Prob	Mascot Ion score	Mascot Identity score	Modifications	Observed	Actual Mass	Charge	Delta AMU	Delta PPM	Retention Time	TIC
(-)TSWLDTK(-)	69%	21.71	43.9014		425.7183	849.4221	2	-0.001258	-1.479	1,886.12	3759660
(-)VFFDmTADGEqLGR(-)	95%	41.67	41.19223	Oxidation (+16), Deamidation (+1)	801.86	1,601.71	2	0.002047	1.277	2,238.79	1162610
(-)VFFDMTADGEQLGR(-)	95%	61.77	41.543327		793.3686	1,584.72	2	-0.001734	-1.093	2,481.68	2352210
(-)HVVFGAIVDGMDVVK(-)	95%	41.22	41.543938		529.2861	1,584.84	3	0.002965	1.87	2,383.40	3269700
(-)VFFDmTADGEQLGR(-)	95%	51.41	41.48078	Oxidation (+16)	801.37	1,600.73	2	0.006063	3.785	2,245.20	828331
(-)VFFDMTADGEQLGR(-)	95%	56.4	41.564884		793.37	1,584.73	2	0.0009781	0.6168	2,489.43	1295000
(-)HTGPGILSmANAGANTnGSQFFITTVK(-)	95%	47.92	38.287888	Oxidation (+16), Deamidation (+1)	917.78	2,750.32	3	-0.01543	-5.608	2,361.08	1140990
(-)HVVFGAIVDGMDVVK(-)	95%	40.28	41.690273		529.28	1,584.82	3	-0.0154	-9.71	2,378.00	4093830
(-)TSWLDTK(-)	77%	26.71	43.95868		425.72	849.4254	2	0.002078	2.444	1,895.10	3589260
(-)VFFDmTADGEQLGR(-)	95%	55.93	41.48078	Oxidation (+16)	801.37	1,600.73	2	0.006063	3.785	2,241.04	1048380
(-)VFFDMTADGEQLGR(-)	95%	80.43	41.54515		793.3694	1,584.72	2	-0.000188	-0.1185	2,492.99	8309720

Cyclophilin A Peptides in Control (aphid proteins and no virus)

Sequence	Prob	Mascot Ion score	Mascot Identity score	Modifications	Observed	Actual Mas	Charge	Delta AMU	Delta PPM	Retention Time	TIC
(-)HVVFGAIVDGmDVVK(-)	64%	16.9	41.627136	Oxidation (+16)	534.6172	1,600.83	3	0.001398	0.8728	2,124.98	2965170
(-)VFFDmTADGEQLGR(-)	95%	79.31	41.168068	Oxidation (+16)	801.36	1,600.71	2	-0.01394	-8.701	2,244.24	2301320
(-)HVVFGAIVDGmDVVK(-)	67%	17.42	41.66104	Oxidation (+16)	534.6159	1,600.83	3	-0.002565	-1.601	2,121.58	7153260
(-)HVVFGAIVDGMDVVK(-)	77%	21.63	41.537537		529.2865	1,584.84	3	0.004213	2.657	2,380.27	1628660
(-)VFFDMTADGEQLGR(-)	95%	52.9	41.564884		793.37	1,584.73	2	0.0009781	0.6168	2,488.48	766518
(-)VFFDmTADGEQLGR(-)	95%	79.06	41.48078	Oxidation (+16)	801.3696	1,600.72	2	0.005291	3.303	2,244.78	1092710
